# Capacity building of primary healthcare providers in Rajasthan, India, for screening and management of common mental health disorders: a study protocol

**DOI:** 10.3389/fpubh.2025.1612961

**Published:** 2025-08-05

**Authors:** Ramesh Kumar Sangwan, Darshana Kansara, Haider Ali, Mukti Khetan, Ramesh Kumar Huda, Vikas Dhikav, Bontha V. Babu

**Affiliations:** ^1^National Institute for Implementation Research on Non-Communicable Diseases, Jodhpur, India; ^2^Department of Health Research, New Delhi, India

**Keywords:** capacity building training, common mental ailments, screening, feasibility, acceptability, effectiveness

## Abstract

**Background:**

Mental disorders impose significant social and financial burdens on individuals, families, and societies, affecting one in seven Indians. Deficient manpower and inadequate training at the graduate level are recognized as poor mental healthcare in India.

**Objectives:**

The study aims to build the capacity of primary healthcare providers, including doctors, nurses and other peripheral health workers in Rajasthan, India. The feasibility, acceptability, and effectiveness of capacity-building training and other activities will be assessed.

**Methods:**

The study will be conducted in four phases: (i) situational analysis, (ii) adaptations of the module and material development and training, (iii) training implementation in the Nagaur district of Rajasthan, and (iv) post-assessment. Initially piloted in the Nagaur district, it will scale up to other districts. Formative research will be conducted with a quasi-experimental pre-post-test design. Existing training modules for Medical Officers, Community Health Officers, General Nurse Midwives, and Auxiliary Nurse Midwives, developed under the Ayushman Bharat Training Manual on Mental, Neurological, and Substance Use Disorders Care, will be adapted. Pre- and post-assessment using structured, validated questionnaires will measure the training’s impact. Quantitative and qualitative data collected at various stages will evaluate capacity-building activities’ feasibility, acceptability, and effectiveness.

**Expected outcomes:**

Doctors are expected to screen and manage common mental health disorders post-training competently. Healthcare providers will be equipped to utilize standardized tools for screening common mental health conditions within primary care settings. This training will enhance the confidence and proficiency of all primary healthcare providers in screening and managing prevalent mental health disorders at the primary care level.

## Introduction

Mental health is a crucial component of overall health, extending beyond the mere absence of mental illnesses. It serves as the cornerstone for individual well-being and optimal functioning, encompassing mental wellness, prevention of mental disorders, treatment, and recovery support (1). Despite the availability of effective prevention and treatment measures, a significant portion of individuals suffering from mental disorders lack access to quality care. Stigma, discrimination, and human rights violations are commonly experienced by many affected individuals (2).

Mental, neurological, and substance use disorders constitute more than 10% of the worldwide disease burden. However, approximately 85% of people in low- and middle-income countries cannot access necessary treatment for these conditions (3, 4). India in 2017 had 197.3 million individuals with mental disorders, constituting 14.3% of the nation’s total population. The greatest burden of Disability-Adjusted Life Years (DALYs) resulting from mental disorders in India that year stemmed from depressive disorders (33.8%) and anxiety disorders (19.0%), followed by DID (10.8%), schizophrenia (9.8%), bipolar disorder (6.9%) and conduct disorder (5.9%) (5).

A global cross-cultural study conducted by the World Health Organization (WHO) across 14 centers revealed that 25% of individuals seeking primary care services were diagnosed with one or more psychiatric disorders. The Indian center of this study, conducted in Bangalore, found that 22.4% of primary care attendees were affected by one or more diagnosable psychiatric disorders (6). India has a treatment gap of up to 83% for any mental disorder, as per the findings of the National Mental Health Survey (7). The treatment gap exists because of inadequate community awareness and a shortage of trained professionals (8). Despite the evidence supporting the integration of mental healthcare into primary care, the goal remains largely unmet in most countries worldwide (9). This shortfall is a significant contributor to the substantial treatment gap experienced by individuals with mental, neurological, and substance use disorders (10).

Approximately 70% of India’s population lives in rural regions (11), while healthcare professionals, including mental health specialists, are predominantly located in urban areas. This mismatch impedes access to services for most of the population (12–17). Incorporating mental health services into primary care settings has the potential to improve accessibility to such services. This integration ensures that mental healthcare is tailored to individuals’ needs, honoring their preferences and upholding safety standards, effectiveness, timeliness, affordability, and quality (18–21).

The primary healthcare workers include doctors (usually allopathic medical graduates), community health officers (either graduates of dental surgery, ayurvedic medicine or nursing), and two categories of health workers. One category is regular health workers (RHWs), and the other is community health workers (CHWs). RHWs include auxiliary nurse-midwives (ANMs) (who have a 2-year diploma in auxiliary nurse-midwifery) and nurses (who have a 3.5-year diploma in general nursing and midwifery or a 4-year graduate degree in nursing) and other trained personnel like health assistants. The CHWs, also called accredited social health activists (ASHAs), are trained community health volunteers with 8 years of formal education and are residents of the village.

Delivering mental healthcare in primary settings faces acknowledged barriers and challenges (22–24). Undergraduate psychiatric training in India is recognized as insufficient. Traditional classroom-based training, which follows a specialist-focused psychiatric curriculum, often lacks practical clinical relevance without additional practical demonstrations. While there have been some digital initiatives in this area, they have been limited in scope and scale (25, 26). For example, this program was conducted at the Telemedicine Centre, Department of Psychiatry, NIMHANS, Bengaluru. The Diploma in Primary Care Psychiatry (DPCP) is a one-year program structured around six core modules. It includes 10 formative assessments based on specific criteria and concludes with a final exit examination. The training framework comprises the following components: a curriculum module centered on the Clinical Schedule for Primary Care Psychiatry; a foundational residential module lasting 10 days conducted onsite; Telepsychiatric On-Consultation Training (Tele-OCT); Virtual Classroom (VCR) sessions; video conferencing-based Continuing Skill Development (V-CSD); Collaborative Video Consultation (CVC); and dedicated modules focusing on public health (25). Another key digital initiative in India, the NIMHANS Digital Academy is a, offering online certificate courses to general physicians, medical officers, and mental health professionals. Covering topics like depression, substance use, and child psychiatry, the platform uses recorded lectures and interactive modules to enable flexible, self-paced learning. It plays a crucial role in addressing workforce gaps and strengthening mental health services at the primary care level (27). Primary care physicians only correctly identify about one-third to half of all disorders. Limited human resources, both in terms of quantity and specialized training in mental healthcare, contribute to this barrier (28, 29).

Primary care physicians are usually the initial point of contact for most patients with psychiatric disorders globally, including in India (25). They often offer symptomatic treatment, which is typically insufficient (25). There is a growing realization that general practitioners, if properly trained, can effectively manage the majority of common mental disorders (28).

Much in the same way, when doctors are being talked about, the situation of the nurses is not very different. One silver lining is that many nursing staff can be trained to provide screening for psychiatric patients, which doctors can deal with after appropriate training. Since primary care is increasingly trusted to implement many national programs, it is relevant that short, concise training could be imparted to Medical Officers, Community Health Officers, General Nurse Midwives, and Auxiliary Nurse Midwives in screening and management of common mental health disorders and burden of psychiatric patients could be reduced by handling patients at primary care level. The same approach to training non-specialists has been tried in low-income and high-income settings (18, 30, 31).

Global initiatives demonstrate the feasibility and effectiveness of integrating mental health services into primary care. For instance, a demonstration project in Nigeria using the WHO’s Mental Health Gap Action Programme (mhGAP) intervention guide showed important improvements in delivering mental health services at the primary care level (32). Similarly, in low-resource countries like Ethiopia, India, Nepal, South Africa, and Uganda, the PRIME project demonstrated the acceptability and feasibility of task-sharing approaches where non-specialist health workers received training to administer mental health care (32). In this regard, similar approaches might be applied toward addressing India’s mental health issues.

In India, training healthcare providers is a longstanding initiative, particularly concerning mental healthcare integration into general healthcare. This principle lies at the heart of the National Mental Health Program and its operational component, the District Mental Health Program (28). Under the *Ayushman Arogya Mandir* initiative, formerly known as Ayushman Bharat- Health and Wellness Centres (AB-HWCs), comprehensive primary care encompasses preventive healthcare and health promotion at the community level, following a continuum of care approach. The services provided at AB-HWCs, including mental health services, are free and universally accessible to all individuals residing within the designated service areas (32). The current study centers on training primary healthcare providers, including medical officers, community health officers, general nurse midwives, and auxiliary nurse midwives, to screen and manage common mental disorders in primary care settings.

This proposal is based on the belief that the identification rate of psychiatric illnesses within general primary care can be significantly improved through the adequate training of Medical Officers in mental ailments. Separate training modules will be developed and delivered to medical officers, community health officers, general nursing midwives, and auxiliary nursing midwives. The training module that will be developed in this initiative stands apart from the standard modules used in other trainings by focusing on context-specific content, skill-based learning, and practical implementation at the primary healthcare level. In contrast to the more generalized format of traditional training, this module will be carefully designed to align with the Ayushman Bharat framework, ensuring a consistent structure, content, and delivery method tailored to different categories of primary care providers.

The study’s objectives are to develop and implement capacity development activities. These activities will include (i) adaptation of the latest training modules (Ayushman Bharat-Health & Wellness centers-2021) for screening and managing common mental ailments in primary care for doctors and other healthcare providers by considering the local context into consideration, (ii) training primary healthcare providers regarding screening and management of common mental health disorders in primary care and (iii) to assess the feasibility, acceptability, and effectiveness of these capacity building activities.

## Methods

Study design: A quasi-experimental pre- post-test design.

Study Duration: 24 Months.

Study Implementation Plan: Adaptation of training modules for simplified screening and management of common mental ailments such as Neurotic disorders (e.g., anxiety), mood disorders (e.g., depression), and substance use disorders. To achieve this, simple, concise, yet comprehensive manuals will be adapted for medical officers, community health officers, general nurse midwives, and auxiliary nurse midwives, which will be useful for screening and managing common mental disorders in primary care. Modules launched under the Ayushman Bharat Training Manual on Mental, Neurological, and Substance Use (MNS) Disorders Care at Ayushman Bharat – Health and Wellness Centres (2021) will be used for adaptation purposes.

An expert group consisting of psychiatrists, clinical psychologists, public health professionals, the state nodal officer (Mental Health), and other health providers will be constituted in the current study to help adapt the content relevant to various cadres of primary healthcare providers. Experts with experience in mental health training, clinical service delivery, public health program implementation, or policy development related to primary healthcare or mental health.

The training material will be administered only after a thorough consultative process and vetting of the content by subject experts. The focus will be to adapt/develop pragmatic and implementable content in the given time frame. To name a few, the same principles/steps mentioned in the training module (2021), e.g., consort for referral and liaison between the Primary Health Centre (PHC)-Medical Officer and District Mental Health Program, will be utilized. Psycho-education, counselling, mental health promotion, and stigma-reducing activities will be included. Also, the same service delivery framework will be utilized.

Apart from adapting the module and training, the focus will be on the third objective, i.e., the implementation research strategy. This strategy aims to assess the training program’s feasibility, acceptability, and effectiveness, aligning with the guidelines outlined in the National Mental Health Policy 2014, sections 4.9 and 4.9.1, emphasizing implementation issues related to mental health in Primary care. The modules will detail disease symptoms, simple and validated tools/scales for screening, diagnosis, aetiology, and practical management tips with appropriate referral indicators.

Phases of the study: The study will be implemented in four stages (Table 1).

**Table 1:** Details of study phases.

Study phases/Activities	Tentative duration	Outcome
PHASE- I Situational analysis Brainstorming session with experts	Six months	Comprehensive understanding of existing training modules, expert-driven refinements, and situational insights to inform the customization of mental health training modules for primary healthcare settings.
PHASE- II •Adaptation of the module and content development Trainings Pilot testing Modification in the modules	Eight months	Development of contextually relevant and user-friendly training modules and materials, validated through expert inputs and pilot testing, ensuring readiness for large-scale implementation.
PHASE- III Training Implementation in Nagaur district of Rajasthan Pre-assessment Training Process	Six months	Effective delivery of training to healthcare providers, with knowledge assessment improving in the management of common mental health disorders.
PHASE- IV •Post assessment •Report writing and publication	Four months	Assessment of effectiveness, feasibility, acceptability of training, long-term impact, and generation of evidence-based recommendations through detailed data analysis and publication of findings.

Phases I and II of the study correspond to Objective (i), Phase III aligns with Objective (ii), and Phase IV addresses the final objective.

### Phase I: situational analysis

Qualitative research will be conducted to understand the challenges and gaps healthcare providers face in delivering mental healthcare at the primary care level and gather information about mental healthcare in the primary care system to contribute to developing a high-quality manual.

Study area: The study will be conducted in different PHCs, CHCs, and HWCs across the different blocks of the Jodhpur district. The study will be conducted in various healthcare facilities across the rural and urban Jodhpur district, Rajasthan.

Study participants: A total of 25 healthcare providers, including medical officers, community health officers, general nurse midwives, and auxiliary nurse midwives, will be selected with their informed consent form using purposive sampling techniques around 15 primary healthcare facilities to gather insights into mental healthcare in primary care settings.

Sampling: A purposive sampling approach, particularly maximum variation sampling, will be utilized to include primary healthcare providers from varied professional and experiential backgrounds related to mental health care. This method is intended to gather a broad spectrum of insights to deepen the understanding of the barriers and enabling factors involved in mental health screening and treatment within primary healthcare settings.

Tools and techniques: A semi-structured interview guide will be used to conduct in-depth interviews with study participants. All the interviews will be transcribed and translated, and thematic analysis for pre-determined themes will be done using an inductive approach. Interviews will be conducted by the research team. Audio recordings of the interviews will be transcribed verbatim and then translated into English by bilingual team members familiar with local dialects and mental health terminology. To ensure rigor in the qualitative phase, following established qualitative research standards will be used including: Triangulation of data sources, independent coding by researchers, followed by reconciliation of differences through discussion, Member checking in select cases to validate key interpretations, Audit trails to document coding decisions and theme development, and Peer debriefing sessions with the study team to ensure credibility and reflexivity.

### Brainstorming session with experts

The expert committee, consisting of public health professionals, psychiatrists, neurologists, state nodal officers, psychologists, scientists, and the research team, will deliberate on recommendations and suggestions. Brainstorming sessions will be conducted to review the material and plan the training. Based on the recommendations from these sessions, extensive research will be conducted on common mental health disorders, the roles and responsibilities of healthcare providers, and mental health capacity-building training.

### Phase II: adaptations of module and material development

The study will adapt training modules developed under the Ayushman Bharat Training Manual on Mental, Neurological, and Substance Use Disorders Care for Healthcare Providers at Ayushman Bharat - Health and Wellness Centers. The training module will be revised to overcome challenges related to cultural relevance, language clarity, and practical application observed during its field use. Major modifications involved tailoring content for specific healthcare cadres, incorporating region-specific examples, simplifying language, and adding hands-on skill-building activities to improve understanding, confidence, and usability in primary care settings. The adaptation will focus on pragmatic and implementable content, including psycho-education, counselling, mental health promotion, and stigma reduction activities.

- *Initial content planning—*

1. A preliminary meeting will be held with Neurologists, psychiatrists, psychologists and Public Health professionals to discuss and outline the content, adaptation process, and timelines.
2. There will be brainstorming sessions to identify and create simple training content.
3. This process will generate a module with a table of contents, followed by a brainstorming meeting to finalize and solidify the contents.

- *Detailed content creation—*The detailed content for each chapter and the overall training structure will be written.
- *Module Validation—*The module’s face and content validity will be assessed in one block of the Nagaur district. The focus will be on ensuring that the trainees grasp the content comprehensively.

### Phase II: trainings

Master trainers, comprising selected subject matter experts, will conduct an initial Training of Trainers (ToT) session. This session will encompass both theoretical content and practical components as detailed in the facilitator’s guide to ensure standardized and effective delivery of the training program across cadres.

### Pilot testing

Pilot testing will be done in one block of Nagaur district, Rajasthan, India. Information will be given to the Primary Health Centres/Community Health Centres in charge, who can nominate Medical Officers, Community Health Officers, General Nurse Midwives, and Auxiliary Nurse Midwives, depending on their nomination. Selected candidates will be given training for a minimum of 1 day.

### Modifications in the module

After the pilot testing, the module will be adapted in consultation with experts such as psychologists, neurologists, psychiatrists, public health professionals and scientists, as stated above.

### Phase-III: training implementation in Nagaur district of Rajasthan

All healthcare providers (MOs, CHOs, GNMs and ANMs) in the Nagaur district will be in the capacity-building training program.

### Pre-assessment

A pre-test will be conducted 1 month prior to the commencement of the training program to assess baseline knowledge. Using structured, validated questionnaires, participants’ baseline Knowledge will be assessed initially (pre-assessment) and at the end of training. The tool is developed by the research team, drawing on the content of the training modules and insights gained from the situational analysis conducted through qualitative research, which will be tailored to the regional context through expert consultations and pilot testing to ensure cultural relevance and reliability.

It will involve the Nagaur district of Rajasthan, where training will be imparted to Medical Officers, Community Health Officers, General Nurse Midwives, and Auxiliary Nurse Midwives. Training related to the topic will be delivered by psychiatrists, clinical psychologists, and/or neurologists as applicable and separately to Medical Officers, Community Health Officers, General Nurse Midwives, and Auxiliary Nurse Midwives from primary care centers and community health centers. Modular training will be given in a phased manner. Practical exercises will be organized at community health centers for the screening of mental health by nurses and doctors, and they will be done under non-communicable disease clinics at community health centers under expert guidance.

Training process- Participation will be voluntary, but to be eligible for a certificate of participation, participants will be required to fill out a Pre-test before the onset of the training and a post-test. Respective healthcare providers are supposed to send quarterly reports of psychiatric patients they see (for 1 year) after the training.

### Phase-IV: post-assessment

The post-assessment test will be administered 1 month after the completion of the training.

Data collection and statistical analysis plan: Data will be collected using pre- and post-assessment questionnaires. The healthcare providers’ scores will be calculated and compared before and after the training is introduced.

Initially, we will assess the distribution of these scores using the Kolmogorov–Smirnov test. The scores follow a normal distribution if the test has a non-significant *p*-value. In such cases, we will report the scores using the mean and standard deviation. However, if there is skewness in the distribution, we will use the median and interquartile range (IQR) to describe the data.

To statistically compare the scores, we will employ either a parametric paired Student’s t-test or a non-parametric Wilcoxon signed-rank test, depending on the data distribution. All the statistical analyses will be conducted using SPSS Software (version 26.0), and a *p*-value equal to or less than 0.05 will be considered statistically significant.

Qualitative data from in-depth interviews, key informant interviews, and focus group discussions will be audio-recorded with the participants’ consent, and field notes will be taken. Data collection will continue until data saturation is achieved, ensuring comprehensive coverage. Qualitative data will be securely stored on password-protected systems. Qualitative data will be analyzed using the thematic analysis, which is best suited exploratory research. All interviews will be cross-referenced with audio recordings and session notes and translated into English; sometimes, back-translations will be performed to ensure accuracy. Standard transcription and translation guidelines will be applied (33, 34). Coding will be done using an inductive approach, with thematic content analysis at six different stages: familiarization with the data, coding, identification of themes, review, definition, and report preparation. The final presentation of the themes and quotations from participants will be used with anonymity (34–36).

Mental health case reporting at the health facilities will be assessed by comparing the number of cases reported before and after the training to evaluate any increase following the intervention. Demographic and clinical diagnostic data sent by healthcare providers every quarter, up to 6 months after training, will be analyzed for diagnostic yield to assess the utilization of training. A simple central web portal will be created to collect data regarding training utilization. The portal will also have videos and training content that can be used for reinforcement training for the reference of medical officers, community health officers, general nurse midwives, and auxiliary nurse midwives, apart from printed modules.

Outcome measurements:

- *Pre- and Post-Assessment Scores:* A before-and-after knowledge assessment for Medical Officers, Community Health Officers, General Nurse Midwives, and Auxiliary Nurse Midwives will be conducted to test the effectiveness of the imparted training.
- *Feasibility:* Feasibility will be assessed by assessing the number of sessions attended (Partial/full) and completed compared to the number of participants enrolled. Key informant interviews will also be conducted with state health officials to identify barriers and challenges.
- *Acceptability:* Acceptability will be assessed by conducting a qualitative evaluation with the participants to identify barriers and enabling factors to adhere to mental health ailment screening & management. A total of 2–3 focused group discussions with 10–12 participants in each group and 2–3 in-depth interviews will be conducted.

The study results will be shared with the program officers of non-communicable disease programs and the national mental health program officers of Rajasthan, India.

Timeline of the study: The timeline for the study is shown in Table 1.

## Discussion

One in eight individuals globally experiences a mental disorder, which is characterized by notable disruptions in thought processes, emotional control, or behavior. Mental and substance use disorders rank among the top causes of disability worldwide. In 2019, depressive and anxiety disorders alone accounted for over 970 million cases globally (37). Mental health is increasingly acknowledged as a key focus in health policies worldwide and has been incorporated into the Sustainable Development Goals (5). In numerous countries, health information systems are not structured to regularly gather data on essential mental health indicators, such as treatment coverage, making it difficult to assess progress (38).

Primary care can effectively manage common mental disorders. Integrating mental health services into primary care is a critical strategy that should be incorporated into national mental health frameworks (39). The integration offers substantial benefits. Firstly, it facilitates early access to necessary mental healthcare services for the entire population, minimizing delays in treatment and ensuring continuity of care. Secondly, providing mental health interventions within primary care settings significantly enhances the likelihood of improved clinical outcomes, including the potential for full recovery, and promotes sustained social integration (40).

The government is actively working to enhance mental healthcare services at the primary care level. Mental health services have been integrated into the Comprehensive Primary Health Care package under the *Ayushman Bharat – Health and Wellness Centres (HWC)* initiative. Additionally, operational guidelines addressing *Mental, Neurological, and Substance Use (MNS) disorders* at HWCs have been issued as part of the Ayushman Bharat framework (41). In 1982, the Government of India launched the National Mental Health Programme (NMHP) with several key objectives. It aims to ensure the availability and accessibility of essential mental healthcare for all, particularly targeting vulnerable and disadvantaged populations. The program also seeks to integrate mental health knowledge into general healthcare and social development initiatives while promoting community involvement in developing mental health services and fostering self-help efforts within communities (16, 42).

Considering the limited availability of mental health specialists, primary care providers (PCPs) are often the first point of contact and must manage the mental health needs of their communities, irrespective of the degree of integration within the healthcare system. For any mental health intervention to take place, a concern must either be reported by the patient or recognized by the PCP during routine care (43). The primary care approach to mental health is grounded in a community-based model that prioritizes mental health promotion, prevention, social engagement, and the organization of health services. It also emphasizes coordination with other sectors and supports individuals’ recovery within their own environments (20, 40).

Strengths: Integrating mental healthcare into the larger healthcare system can enhance service coordination, leading to improved outcomes for individuals facing both physical and mental health issues. The Ayushman Bharat initiative has the potential to invest in training healthcare providers, particularly primary care physicians, to identify and manage common mental health conditions, thereby addressing the shortage of mental health specialists in the country. Moreover, the program can collect data on mental health service utilization and treatment outcomes, providing valuable insights to inform future policy decisions and optimize resource allocation for mental health services (44).

Limitations: Some Medical Officers, Community Health Officers, General Nurse Midwives, and Auxiliary Nurse Midwives may be unable to attend the training program due to unavoidable circumstances.

Expected outcomes: A regionally adapted, evidence-informed training module—aligned with the Ayushman Bharat-HWC (2021) framework—will be developed and implemented to enhance the knowledge, skills, and confidence of primary healthcare providers in the identification and management of common mental health disorders. The intervention will be tailored to local needs to improve contextual relevance and service delivery at the primary care level. The study will also evaluate the feasibility, acceptability, and effectiveness of this capacity-building approach to support its potential integration into routine primary healthcare systems.

Future plans based on expected outcomes: To enhance the sustainability of the training, the health system will be involved from the outset of the study to facilitate their integration as a standard component of the Ayushman Bharat capacity-building initiatives. If the training proves effective, it would spread across all States and Union Territories and form an integral component of the District Mental Health Programme. The national and state health authorities will be encouraged to develop partnerships to institutionalize mental health training into existing healthcare frameworks, thereby ensuring a long-term impact on improving mental health delivery at the primary care level. Uniform modules will be developed to ensure consistency in training delivery across districts and will be used to train healthcare providers. Standard Operating Procedures (SOPs) will be established to ensure uniformity in the implementation and evaluation of training across all regions.

## Data Availability

The original contributions presented in the study are included in the article/supplementary material, further inquiries can be directed to the corresponding author/s.
