# Correction: Capacity building of primary healthcare providers in Rajasthan, India, for screening and management of common mental health disorders: a study protocol

**DOI:** 10.3389/fpubh.2025.1682116

**Published:** 2025-08-12

**Authors:** Frontiers Production Office

**Affiliations:** Frontiers Media SA, Lausanne, Switzerland

**Keywords:** capacity building training, common mental ailments, screening, feasibility, acceptability, effectiveness

In the acknowledgments, the incorrect information was present. The correct Acknowledgments appears below:

“We acknowledge the Indian Council of Medical Research, New Delhi, for funding this study. The entire study will be executed in collaboration with the state government of Rajasthan.”

The original version of this article has been updated.

